# Exosomes in the Chemoresistance of Glioma: Key Point in Chemoresistance

**DOI:** 10.1111/jcmm.70401

**Published:** 2025-02-14

**Authors:** Xu Guo, Haozhe Piao, Rui Sui

**Affiliations:** ^1^ Department of Neurosurgery Cancer Hospital of Daflian University of Technology, Liaoning Cancer Hospital & Institute Shenyang Liaoning China

**Keywords:** chemoresistance, exosomes, glioma, ncRNAs, tumour microenvironment

## Abstract

Gliomas are the most ordinary primary virulent brain tumours and commonly used clinical treatments include tumour resection, radiation therapy and chemotherapy. Although significant progress has been made in recent years in progression‐free survival (PFS) and overall survival (OS) for patients with high‐grade gliomas, the prognosis for patients remains poor. Chemoresistance refers to the phenomenon of decreased sensitivity of tumour cells to drugs, resulting in reduced or ineffective drug efficacy, and is an important cause of failure of tumour chemotherapy. Exosomes, a type of extracellular vesicle, are secreted by cancer cells and various stromal cells in the tumour microenvironment (TME) and transfer their inclusions to cancer cells, increasing chemoresistance. Furthermore, depletion of exosomes reverses certain detrimental effects on tumour metabolism and restores sensitivity to chemotherapeutic agents. Here, we summarised the correlation between exosomes and resistance to chemotherapeutic agents in glioma patients, the mechanisms of action of exosomes involved in resistance and their clinical value. We aimed to afford new thoughts for research, clinical diagnosis and intervention in the mechanisms of chemoresistance in glioma patients.

**Abbreviations**
BBB: blood–brain barrier
CAFs: cancer‐associated fibroblasts
ciRNA: intron circular RNA
DUSP3: double specificity phosphatase 3
ECM: extracellular matrix
EMT: epithelial‐mesenchymal transition
ERK: extracellular signal‐regulated kinase
EV‐miR‐30b‐3p: ev‐packaged miR‐30b‐3p
exo: exosomes
GBM: glioblastomas
HGF: hepatocyte growth factor
HGG: high‐grade gliomas
IL‐6: interleukin 6
MDSCs: marrow‐derived suppressor cells
MGMT: O6 methylguanine DNA methyltransferase
ncRNAs: noncoding RNAs
NHA: normal human astrocytes
OLFML3: olfactomedin‐like 3
OS: overall survival
PFS: progression‐free survival
TAMs: tumour‐associated macrophages
TGF‐β: transforming growth factor‐β
TME: tumour microenvironment
TMZ: Temozolomide
TNF‐α: necrosis factor‐α
WHO: World Health Organisation
XRCC4: X‐ray restore complementation 4

## 1 Introduction

Gliomas are originated from undifferentiated glial or neural progenitor cells and are the most usual elementary tumours of the nuclear nervous system, characterised by invasive growth, rapid progression, difficulty in complete resection, frequent recurrence and poor prognosis [1, 2]. The aetiology of glioma is not entirely understood, and genetic opportunities and ionising radiation are deemed to be causative factors [3, 4]. Based on the World Health Organisation (WHO) standard, low‐grade gliomas (LGG) comprise WHO grades 1 and 2, high‐grade gliomas (HGG) comprise WHO grades 3 and 4, and glioblastomas (GBM) comprise the majority of WHO grade 4 gliomas and are the most lethal and recurrent [5]. GBM comprises the majority of WHO grade 4 and is the most lethal and recurrent [5, 6]. Current clinical treatments for glioblastoma involve surgical resection, radiation treatment, chemotherapy, molecular targeted treatment and supportive care [7, 8, 9, 10, 11], but patient prognosis remains poor. However, patient prognosis remains bad, with a median subsistence time for gingival GBM of only about 1 year and a 5‐year survival rate of no more than 10% [12, 13]. Temozolomide (TMZ), the first‐line chemotherapeutic agent used in combination with postoperative radiation therapy, is still insufficient to improve prognosis, and patients eventually develop TMZ resistance [14, 15]. Other targeted therapies or combinations of therapies for glioma have not made significant progress: tyrosine kinase inhibitors targeting the EGFR alone or in combination with TMZ failed to significantly improve prognosis [16]. pI3K and mTOR inhibitors and vascular endothelial growth factor inhibitors were similarly ineffective [17, 18]. Therefore, exploring the mechanisms of glioma resistance to chemotherapy is critical to improving glioma outcomes and is a current focus of basic glioma research.

Exosomes are nano‐sized extracellular microparticles encased in a lipid bilayer of 30–150 nm, derived primarily from the multivesicular body and released into the extracellular surrounding upon fusion of the multivesicular body with the cell membrane [19, 20]. Exosomes are secreted by almost all cell kinds, tumour cells are included, and are present in supernatants of cultured cells and in different kinds of body fluids, including blood, saliva, urine, breast milk, cerebrospinal fluid and amniotic fluid [21, 22]. Exosomes contain various types of molecules, lipids, proteins are included, DNA, mRNA and noncoding RNA, and by transporting and transporting these substances, they play a crucial character in the exchange of information between cells and in functional changes in the recipient cells [23]. Thus, exosomes can exchange products with other cells by mediating specific intercellular communication, eliminate unwanted outcomes from cells and activate signalling pathways in fusing or interacting cells [24, 25]. Studies have shown that exosomes are involved in the constitution and advancement of a variety of cancer processes, reconstruction of the tumour microenvironment, angiogenesis, immune flee, dissemination and metastasis are included [26, 27]. Tumour cell‐derived exosomes may interact with immune cells in the tumour microenvironment to aid in tumour cell immune escape and improve tumour advancement and therapeutic obstruction in cancer cells [28, 29]. In late years, the study of exosomes in tumour therapy and drug resistance has become an emerging hotspot.

This paper outlined the mechanisms of exosomes involved in glioma drug resistance and their value in targeted therapy, and provided new ideas for future research on targeted therapy of glioma.

## 2 Overview of Exosomes

### 2.1 The Biogenesis and Biological Properties of Exosomes

**FIGURE 1:**
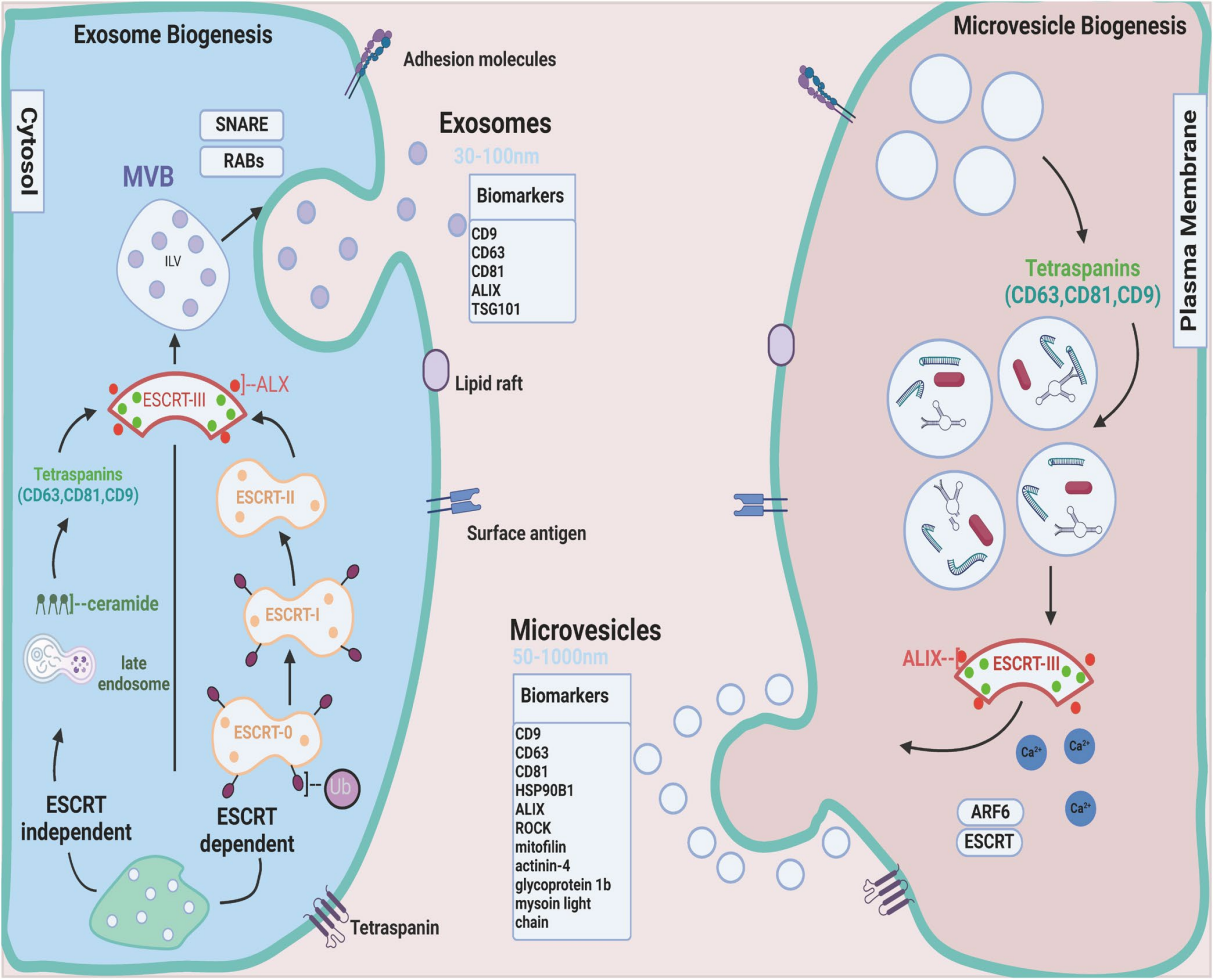
The biogenesis of exosomes and microvesicles. Exosomes exert various cellular functions through intercellular communication. Exosome biogenesis involves endocytosis and the formation of multivesicular bodies (MVBs). Exosomes are indented inwardly by endocytosis in the mother cell to form MVBs. MVBs contain multiple intraluminal vesicles (ILVs), which are fused to specific parts of the cell membrane of the mother cell and are released outside the cell as exosomes in the form of vesicular exocytosis. Microvesicles are secreted from the plasma membrane by vesiculation and release from the plasma membrane, and their shedding can be driven by different stimuli (e.g. increase in intracellular calcium ions). The process of microvesicle formation is relatively simple, with outward bud secretion from the cell membrane into the extracellular matrix, and its biogenesis is achieved mainly by altering the distribution of phospholipids within the plasma membrane and the contractility of cytoskeletal components.

Cells of various organisms, including all eukaryotes and some prokaryotic cells, can release vesicles into the extracellular environment [30]. Exosomes are a type of small extracellular vesicle with nucleotidase activity and 40–100 nm in diameter [20, 31]. The endocytosis pathway during exosome formation is completed by a highly dynamic membrane involved in internalisation of extracellular ligands and cellular components, which are then recycled or degraded toward the plasma membrane. Early endosomes mature into late endosomes, which accumulate in the lumen during the process. Because of their morphological characteristics, they are often referred to as multivesicular endosomes or multivesicular bodies. In most cells, the multivesicular bodies fuse with lysosomes and are precisely degraded. However, multivesicular bodies with certain characteristics, including CD63, a lysosome‐associated membrane protein, and other proteins normally found in recent endosomes, can also fuse to the plasma membrane and release their substances into the extracellular environment [32, 33, 34]. Exosomes involve cell‐specific proteins, lipids and nucleic acids that can act as signalling molecules to other cells and vary their function [35, 36]. The cell membrane of exosomes is sufficient in cholesterol, sphingolipids, ceramides, lipid rafts and phosphatidylserine, which protect internal proteins like cytokines and growth factors and biologically active substances such as lipids, coded and non‐coded RNA from regression and dilution in the extracellular surrounding and facilitates the long‐distance conveyance of these matters through tissue fluids and blood flow. Ligands allow efficient binding to receptor cells [37, 38]. The biogenesis of exosomes and microvesicles was displayed in Figure 1.

### 2.2 Challenges in Exosome Isolation and Standardisation of Protocols

Exosomes can be isolated from various biological fluids, including blood, cerebrospinal fluid (CSF) and cell culture supernatants. However, the techniques used for exosome isolation vary widely. Common methods include ultracentrifugation, size exclusion chromatography (SEC), density gradient centrifugation and immunoaffinity capture [39, 40, 41, 42]. Each method has its own advantages and limitations, particularly in terms of yield, purity and time efficiency. Ultracentrifugation, while the most widely used, often results in contaminants such as proteins or larger vesicles, which can affect the accuracy of subsequent analyses. Size exclusion chromatography offers a more gentle method with less potential for contamination, but it often yields smaller amounts of exosomes, which can be a limitation for high‐throughput studies or clinical applications.

Moreover, exosome quantification remains a significant challenge. While nanoparticle tracking analysis (NTA) and dynamic light scattering (DLS) are commonly used to determine the size and concentration of exosomes [43, 44], these methods can be imprecise, particularly when working with heterogeneous populations of exosomes. Additional techniques such as western blotting for specific exosomal markers (e.g. CD63, CD81 and TSG101) and electron microscopy for visual confirmation are also employed [45], but these require substantial technical expertise and can be resource‐intensive.

To address these challenges, there is a growing need for the standardisation of exosome isolation protocols, including clear guidelines on sample collection, processing and storage [46]. Furthermore, the development of more robust and automated methods for exosome isolation and quantification could greatly enhance the reproducibility and reliability of exosome‐based research. Standardisation would not only ensure more consistent results across different research groups but also pave the way for clinical adoption, where precision and reproducibility are critical. The isolation and standardisation methods of exosomes were displayed in Table 1.

**TABLE 1:** The isolation and standardisation methods of exosomes.

Method	Description	Advantages	Disadvantages	Challenges for standardisation
Ultracentrifugation	High‐speed centrifugation to pellet exosomes from biological fluids (e.g. blood, CSF).	Widely used, relatively simple, can process large volumes.	Time‐consuming, potential contamination with other vesicles and proteins, requires expensive equipment.	Standardising speed, time, and temperature settings; reducing contamination.
Density Gradient Centrifugation	Uses density gradients (e.g. sucrose gradients) to separate exosomes based on their density.	Higher purity compared to ultracentrifugation.	Time‐consuming, complex setup, requires expertise, potential loss of exosomes.	Standardising gradient composition and centrifugation conditions.
Size Exclusion Chromatography (SEC)	Separation based on size using chromatographic columns.	Gentle on exosomes, reduces contamination, can be automated.	Lower yield, requires specialised columns and expertise, smaller sample volumes.	Standardising column materials, flow rates, and buffer conditions.
Immunoaffinity Capture	Uses antibodies against exosome surface markers (e.g. CD63, CD81) to capture exosomes from a mixture.	High specificity, can isolate specific exosome subtypes.	Expensive, limited to known surface markers, potential loss of exosomes, requires specialised reagents.	Standardising antibody types, concentrations, and binding conditions.
Polymer‐Based Precipitation	Uses polymers (e.g. PEG) to precipitate exosomes from biological fluids.	Simple, does not require specialised equipment, can process large volumes.	Potential contamination with other proteins and vesicles, variable efficiency, difficult to remove polymer residue.	Standardising polymer concentrations, incubation times, and purification steps.

Finally, improvements in exosome characterisation are essential to fully realise their potential in therapeutic applications. The complexity of exosome cargo—ranging from proteins to RNAs—requires a multi‐faceted approach to profiling. Advances in high‐throughput sequencing and proteomics will be crucial in fully characterising exosome contents, which will, in turn, improve their clinical utility, particularly for personalised medicine in glioma treatment.

### 2.3 The Biological Function of Exosomes in Cancers

As research on exosomes continues to advance, the role of exosomes in tumour pathological processes continues to be uncovered. Exosomes secreted by tumour cells can improve the formation of the tumour microenvironment (TME) and promote cancer cells avoid immune supervision and tumour cell growth [47, 48]. Besides, exosomes secreted by tumour cells can also induce neovascularization, thereby ensuring access to nutrition and helping tumour cells continue to proliferate [49, 50]. Tumour tissue is composed of tumour cells and several types of stromal cells, creating a specific microenvironment for tumour growth. Fibroblasts are the main component of stromal cells and are the main source of collagen and other extracellular matrix (ECM)‐related proteins [51]. When activated by exosomes, fibroblasts differentiate into cancer‐associated fibroblasts (CAFs), leading to collagen over‐deposition and ECM remodelling [52, 53]. The initial impact of cancer‐associated fibroblasts on tumour development depends primarily on the abnormal secretion of various proteins from exosomes, hepatocyte growth factor (HGF), tumour necrosis factor‐α (TNF‐α), interleukin 6 (IL‐6) and transforming growth factor‐β (TGF‐β) are included. Exosomes are tumour cell‐derived proteins that carry immunosuppressive molecules and directly or indirectly transmit these substances to immune cells, inhibiting immune cell function and promoting tumour cell migration and proliferation [54, 55]. Tumour angiogenesis, growth and metastasis require blood vessels to supply oxygen and nutrients, and exosomes are also involved in the regulation of pathological angiogenesis [56, 57]. Exosomes are involved in the epithelial‐mesenchymal transition (EMT) process, conveying mesenchymal‐related feedback between tumour cells and the microenvironment and adjusting signal transduction in receptor cells [36, 58].

## 3 Exosomes Can Regulate the Microenvironment of Glioma

The glioma microenvironment is consisted of a variety of cells, containing tumour cells, perivascular cells, extracellular matrix, intrinsic immune cells, T lymphocytes, neurons and astrocytes [8, 59, 60]. In particular, exosomes derived from glioma cells can promote tumour cell growth by affecting other cells, thereby inducing changes in the properties of the tumour cells themselves [61, 62, 63]. Gliomas interact with surrounding noncancerous cells and maintain a microenvironment conducive to tumour growth, tumour invasion, angiogenesis, immunosuppression and tumour drug resistance [64, 65]. Multiple modes of transduction are involved in this process, including soluble factors and gap junctions. Biomolecules transported and released by tumour cell exosomes include not only soluble proteins, but also a variety of coding and non‐coding RNAs and proteins that change gene presentation in recipient cells, induce phenotypic changes in recipient cells and extracellular matrix remodelling, and contribute to tumour cell growth [66, 67].

### 3.1 Angiogenesis

In the glioma microenvironment, exosomes can regulate multiple processes like tumour cell diffusion, subsistence and migration by adjusting angiogenesis in the tumour microenvironment. The glioma stem cell‐derived exosome miR‐26a promotes angiogenesis in glioma microvascular endothelial cells [68]. miR‐9 improves tumorigenesis and angiogenesis in human gliomas and is drived by MYC and OCT4 [69]. Exosomal microRNA‐148a‐3p promotes tumour angiogenesis by inhibiting ERRFI1 activation of the EGFR/MAPK signalling pathway in glioma [70]. Glioma cells can eliminate miR‐204‐3p inhibition by upregulating SUMOylation to promote angiogenesis under hypoxic conditions, making the SUMOylation inhibitor TAK‐981 a possible glioma therapeutic agent [71]. Angiogenesis is one of the fundamental processes for tumour growth. Exosomes regulate angiogenesis through multiple signalling pathways (such as the PI3K/AKT, MAPK and JAK/STAT pathways), providing the necessary nutritional supply to the tumour microenvironment and accelerating tumour progression. Exosomal miRNAs play a critical role by directly or indirectly regulating these key signalling pathways.

### 3.2 Tumour Metastasis

There are several related mechanisms of exosome action in tumour metastasis, including intercellular signalling, changes in the tumour microenvironment, cell adhesion and migration. Studying the mechanism of action of exosomes in tumour metastasis can identify new drug targets and therapeutic strategies to inhibit tumour metastasis or improve therapeutic efficacy. Exosomal circARID1A can participate in regulating the migration and invasion of GBM by regulating miR‐370‐3p/TGFBR2 [72]. Exosome‐mediated conveyance of circWDR62 can improve TMZ obstruction and virulent tumour growth and metastasis by targeting the glioma miR‐370‐3p/MGMT axis in vitro and in vivo [73]. Additionally, miRNAs and circRNAs carried by exosomes play a vital role in tumour cell migration and metastasis. They regulate different signalling pathways and target genes, not only promoting the spread of tumour cells but also enhancing tumour drug resistance.

### 3.3 Tumour Immunity

Immune cells and associated cytokines in the tumour microenvironment play a notable part in tumour progress and advancement. Studies have suggested that tumour cell‐derived exosomes have anti‐tumour immune effects, primarily through direct inhibition of immune cells or by regulating the expression of associated cytokines that promote tumour immune escape, and play an crucial part in tumour progress and advancement. The tumour‐associated macrophage‐derived exosome LINC01232 induces immune escape in gliomas by downregulating surface MHC‐I expression [74]. Exosome miR‐1246 in glioma patient fluid could promote MDSCs disaffinity and activation in a double specificity phosphatase 3 (DUSP3)/extracellular signal‐regulated kinase (ERK)‐dependent manner [75]. miR‐1298‐5p was enriched in CSF exosomes and inhibited glioma advancement in in vitro and in vivo experiments. miR‐1298‐5p promotes glioma development by improving the immunosuppressive effects of bone marrow‐derived suppressor cells (MDSCs) [76]. rBP‐J OE Co‐culture and overexpression of circBTG2 in Mφ‐Exos inhibited glioma cell growth and invasion, whereas circBTG2‐knockdown promoted tumour growth in vivo [77]. Exosomes are also involved in regulating tumour immune escape and the tumour immune microenvironment, which may enhance the tumour cells' resistance to immunotherapy through interactions with various immune pathways (such as JAK/STAT, PI3K/AKT, etc.).

## 4 Exosomes Are Involved in Chemoresistance of Glioma in Distinct Cells

**FIGURE 2:**
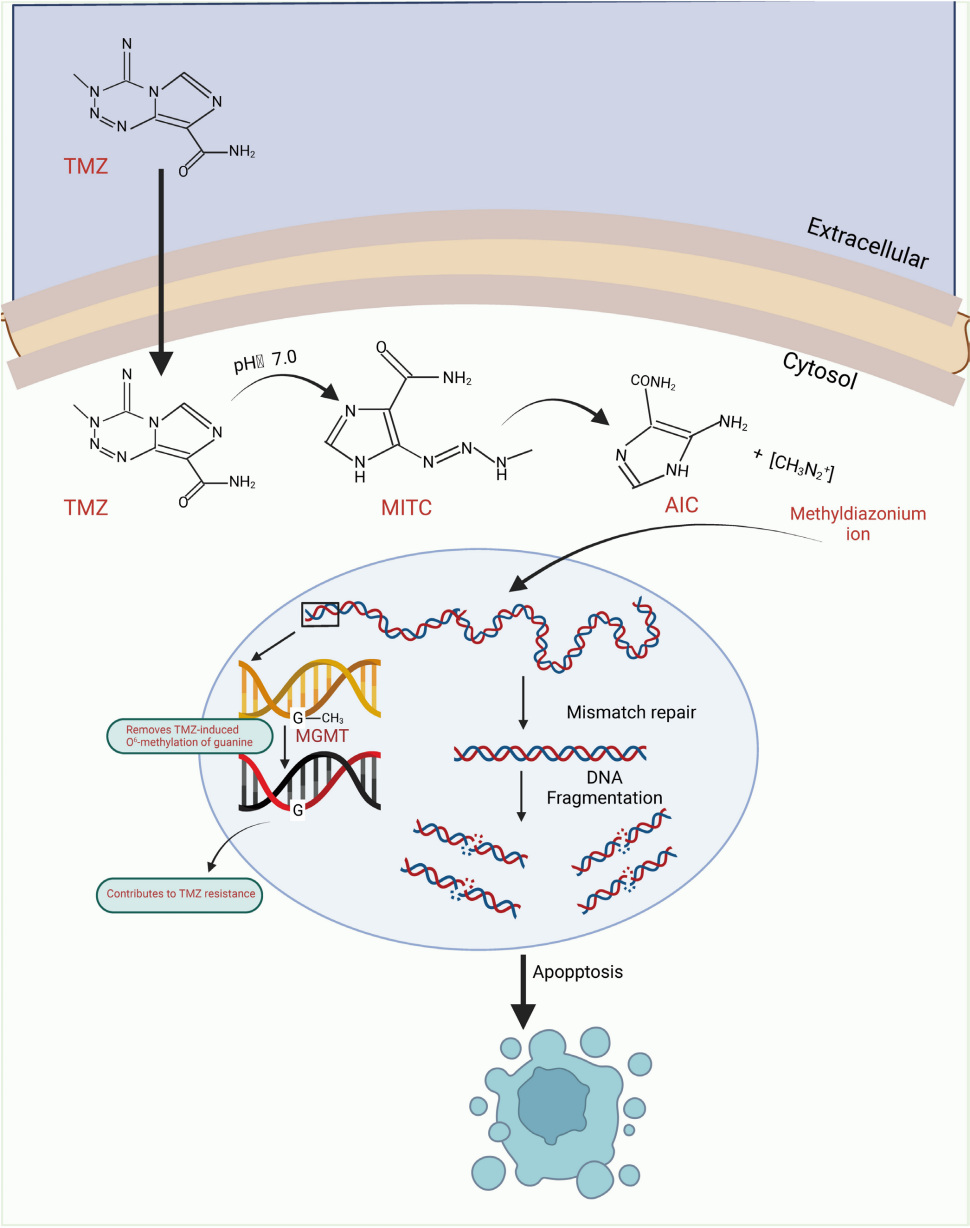
The potential mechanism of temozolomide (TMZ). The primary mechanism of action of temozolomide is to cause cell death by damaging the DNA structure of tumour cells. It is an alkylating agent capable of intensifying the DNA damage response, leading to DNA instability, which in turn causes multiple forms of cell death within tumour cells. Briefly, in the cytoplasm TMZ can be converted to MTIC to produce methyl diazo cations that transfer their methyl groups to the N7 and O6 sites of guanine and to the N3 site of adenine. TMZ adducts confer mutations in DNA immobilised by O6 methylguanine‐DNA methyltransferase (MGMT), mismatch repair (MMR), and base excision repair (BER) in TMZ‐sensitive cells produced DNA double‐strand breaks (DSBs) and triggered programmed cell death.

Exosomes may be involved in the generation and spread of drug resistance by carrying and transporting substances such as drug resistance‐associated proteins, genes and compounds [78]. Drug resistance‐associated proteins and genes are passed from cell to cell with the help of exosomes, which may lead to the spread and inheritance of drug resistance [79, 80]. These exosomes may originate from tumour cells that produce drug resistance or from other cells in the surrounding drug‐resistant microenvironment. Studies have shown that the drug resistance of cells can be influenced by modulating the release and content of exosomes [81, 82]. Certain drugs can inhibit the production of exosomes or alter their contents, thereby reducing cellular resistance. TMZ is currently the first‐line clinical chemotherapeutic agent and is a new type of alkylating agent with an imidazolium tetrazine ring that crosses the blood–brain barrier very easily, with oral bioavailability reaching 100%, and a new type of It is an alkylating agent and is widely used because it reaches 100% oral bioavailability, crosses the blood–brain barrier very easily, and has few clinical side effects [83, 84]. However, TMZ resistance seriously affects the efficacy of TMZ chemotherapy in the treatment of glioma patients, a problem that needs to be urgently resolved in the treatment of glioma [85, 86]. Previous studies have shown that TMZ resistance in gliomas is the result of a variety of mechanisms and factors, involving a variety of regulatory molecules. Current studies indicate that possible molecular mechanisms of TMZ resistance in gliomas are mainly DNA damage repair, abnormally high expression of multidrug resistance‐related proteins, altered apoptotic signalling pathways, tumour stem cell mediation and alterations in the tumour immune microenvironment [87, 88, 89] (Figure 2).

Research has shown that different cells can regulate chemoresistance in gliomas by transmitting exosomes (Table 2). Wang et al. [90] found that R‐EXO‐T/D has multiple advantages, including nano‐embryo size with better blood–brain barrier (BBB) permeability, accumulation of tumour‐localised homologous impacts and boosted antitumor activity that blocks TMZ resistance and induces immune answer. Zeng et al. [91] found that phosphorylated MET is only detected in the PTPRZ1‐MET fusion gene (ZM fusion gene) ZM exosomes. ZM exosomal gene expression was altered and not only induced epithelial‐mesenchymal transition in non‐ZM fusion GBM cells and usual person astrocytes, but also decreased an exosome‐dependent phenotype defined by GBM cell transfer and aggression, neurosphere proliferation and angiogenesis and in GBM cells conferred TMZ obstruction effectors. These results suggest that exosomes mediate GBM invasiveness and that ZM fusion exacerbates this effect. Yu et al. [92] adxised that normal human astrocytes (NHA) are transformed into response astrocytes (RAS), that the ratio of O6 methylguanine DNA methyltransferase (MGMT) mRNA to non‐reactive NHA is significantly increased in exosomes (EXO) released from the RAS, and that RAS‐EXO is MGMT passive glioma cells, and a TMZ resisted phenotype are obtained in vitro and in vivo by interpretation of exogenous exosomal MGMT mRNA. This mechanism suggests that MGMT‐negative glioma cells can be spared from TMZ‐induced apoptosis by functional intercellular transfer of glioma‐associated NHA via exosomal MGMT mRNA. Yu et al. [93] found low expression of MiR‐199a but high expression of AGAP2 in glioma tissues and cells. Exosome‐mediated MSC delivery of miR‐199a to glioma cells inhibited glioma cell diffusion, aggression and transfer. Overexpression of miR‐199a by MSCs gave rise to a prominent rise in chemosensitivity to TMZ and suppressed tumour rise in vivo. MSC‐derived exosomal miR‐199a downregulates AGAP2 and inhibits glioma progression. Yin et al. [94] found that EV‐packaged miR‐30b‐3p (EV‐miR‐30b‐3p) could target RHOB and leads to reduced apoptosis and raised diffusion in vitro and in vivo. This mechanism suggested that miR‐30b‐3p in CSF or as a possible biomarker to predict TMZ obstruction, and targeting EV‐miR‐30b‐3p was a underlying therapeutic strategy for GBM. Li et al. [95] found that exosomes take up lnc‐TALC and transport it to tumour‐associated macrophages (TAMs), where it acts as a promoter of M2 polarisation in microglia. c5 promotes restore of TMZ‐induced DNA injure causing chemotherapy rejection, while enhanced efficacy of C5 a‐targeted immunotherapy acts as a limiting effect on lnc‐TALC‐mediated TMZ resistance. The mechanism by which lnc‐TALC delivered by exosomes remodels the GBM microenvironment and renders tumours less sensitive to TMZ chemotherapy was demonstrated, suggesting that lnc‐TALC‐mediated crosstalk between GBM cells and microglia plays an inhibitory role in the efficacy of chemotherapy. Wang et al. [96] found that HOTAIR in GBM cells competitively binds to miR‐526b‐3p, significantly reducing the ability of miR‐526b‐3p to bind EVA1 and increasing EVA1 expression. HOTAIR encapsulating serum EVs significantly increased tumour raise and TMZ obstruction in vivo by attenuating miR‐526b‐3p‐mediated suppression of EVA1. GBM‐serum EV‐encapsulated HOTAIR, or miR‐526b‐3p downregulation and upregulation of EVA1 enabled a significant increase in GBM progression and chemotherapy resistance via upregulation of EVA1. Yang et al. [97] found that knockdown of PTRF increased proliferation and significantly decreased apoptosis of GBM cells after TMZ treatment. Increased efflux of TMZ by PTRF via EVs resulted in a significant increase in TMZ resistance. CQ therapy after sequential TMZ treatment effectively improved the efficacy of TMZ against GBM due to increased intracellular TMZ concentration ([TMZ]i). Improvement. Wu et al. [98] found that exosomes originated from hypoxic glioma cells improved propagation and inhibited apoptosis of glioma cells treated with TMZ, which was inhibited by the miR‐106a‐5p inhibitor TMZ increased PTEN and Bax levels and attenuated p‐Akt levels in glioma cells TMZ increased PTEN and Bax levels and attenuated p‐Akt levels in glioma cells. Exosomal miR‐106a‐5p in hypoxic glioma cells was shown to significantly lessen the susceptivity of glioma cells to TMZ by downregulating PTEN. Liu et al. [99] showed that circCABIN1 can regulate the presentation of olfactomedin‐like 3 (OLFML3) via spongy miR‐637. Elevated expression of OLFML3 activated the ErbB signalling pathway, ultimately resulting in stem reprogramming and TMZ obstruction. circCABIN1 and OLFML3‐targeted artificial exosomes in GBM ectopic mice significantly increased TMZ targeting and antitumor activity.

**TABLE 2:** Potential roles and mechanism of exosomes in the regulation of chemoresistance in glioma.

Molecular	Parent cell/source	Target cell	Target	Biological function	Reference
MET	ZM Fusion GBM Cells	Non‐ZM fused GBM cells	\	Promote cell migration and invasion, neurosphere growth and angiogenesis, and TMZ resistance	[83]
MGMT	Astrocyte	MGMT negative glioma cells	\	Promote TMZ resistance	[84]
miR‐199a	MSCs	glioma cells	miR‐199a/AGAP2	Inhibit cell proliferation, invasion, and migration, tumour growth in vivo, and enhance chemotherapy sensitivity of TMZ	[85]
miR‐30b‐3p	GSCs	glioma cells	HIF1α/STAT3/miR‐30b‐3p/RHOB	Promote cell proliferation, inhibiting cell apoptosis, and enhance TMZ resistance	[86]
lnc‐TALC	GBM cells	TAMs	lnc‐TALC/ENO1/p38/MAPK	Promote M2 polarisation of microglia and enhance TMZ resistance	[126]
HOTAIR	Serum	GBM cells	HOTAIR/miR‐526b‐3p/EVA1	Promote cell proliferation, invasion, tumour growth and enhance TMZ resistance	[88]
miR‐106a‐5p	Hypoxic glioma cells	glioma cells	miR‐106a‐5p/PTEN/Bax/p‐Akt	Promote cell proliferation, inhibiting cell apoptosis, and enhance TMZ resistance	[90]
circCABIN1	TMZ resistant cells	GSCs	circCABIN1/miR‐637/OLFML3	Promote stemness reprogramming and TMZ resistance	[91]

## 5 Exosomes Are Involved in the Transmission of Chemotherapy Resistance in Gliomas

During tumour development, the degree of heterogeneity varies, that is, different tumour cells in the same tumour tissue have different sensitivities to chemotherapeutic agents. Recently, it is becoming more clear that exosomes also play a crucial part in the exchange of information between tumour cells. Tumour cells can be classified into resistant and sensitive cells according to their sensitivity to chemotherapeutic agents, and discrepancies in sensitivity of tumour cells to chemotherapeutic agents can be transmitted from cell to cell by this mechanism, causing the procurement of resistance in sensitive cells [100, 101]. Research has shown that chemotherapy resistant glioma cells can also regulate the resistance of chemotherapy sensitive cells by delivering exosomes (Table 3). Yang et al. [102] discovered that miR‐221 expression was dramatically upregulated in both glioma tissues and exosomes. suppression of miR‐221 expression in SHG‐44 cells notably constrained cell propagation, transfer and TMZ obstruction, while treatment of SHG −44 cells, treatment promoted their malignant transformation. In conclusion, the RELA/miR‐221/DNM3 regulatory axis is a potential diagnostic and treated aim for glioma. Zeng et al. [103] found that restoring miR‐151a expression inhibited XRCC4‐mediated DNA restore, giving rise to increased sensitivity of TMZ‐resistant GBM cells. TMZ chemotherapy obstruction in recipient TMZ‐sensitive cells was conferred by TMZ‐resistant GBM cells in an exosomal miR‐151a deletion‐dependent pattern. Restoration of exosomal miR‐151a in donor TMZ‐resistant cells removed the spread of chemotherapy resistance by donor TMZ‐resistant cells. The miRNA profiles of CSF‐derived exosomes reflected the potential chemotherapy resistance status of GBMs based on the presentation level of miR‐151a. Yin et al. [104] found that TMZ resistance was propagated by the process of uptake of biologically active miR‐1238 in exosomes shed from TMZ‐resistant cells into TMZ‐sensitive cells. Exosomal miR‐1238 may afford chemotherapy resistance in the tumour microenvironment. These outcomes indicate that circulating miR‐1238 is a clinical biomarker and a hopeful therapeutic aim for TMZ obstruction in GBM. Zhang et al. [105] found that SBF2‐AS1 acts as a ceRNA for miR‐151a‐3p and inhibits its endogenous target, X‐ray restore complementation 4 (XRCC4), resulting in enhanced DSB repair in GBM cells. High levels of SBF2‐AS1 in exosomes of TMZ‐resistant GBM cells allowed TMZ resistance to spread in GBM cells undergoing chemotherapy GBM cells enriched exosomes by secreting oncogenic lncSBF2‐AS1, which reorganises the tumour microenvironment and upgrade chemotherapy rejection in GBM cells. Yang et al. [106] found that (Dio)‐stained exosomes are taken up by glioma cells, that uptake of Dio‐stained rExo by U251s cells is more pronounced than that of Dio‐stained sExo, and that Gap27 attenuates cellular uptake of rExo43. rExo significantly increased IC50 values, colony formation and Bcl‐2 expression in U251s cells in response to TMZ, but caused significant increases in Bax and cleaved caspase‐3 expression. Caspase‐3 expression was decreased. rExo enhanced U251s cell migration, while Gap27 inhibited cell migration by rExo. Wang et al. [107] showed that A172R‐derived exosomes could promote proliferation and TMZ resistance in susceptible GBM cells, that depletion of exosomal miR‐25‐3p partially inhibited the effects induced by exosome transfer in A172R cells, and that overexpression of miR‐25‐3p caused a prominent raise in proliferation and TMZ rejection in susceptible GBM cells. knockdown of FBXW7 promoted proliferation and TMZ resistance in GBM cells. Wei et al. [108] found that exosomal MIF induced TMZ obstruction in sensitive cells by enhancing cell diffusion and constrainning apoptosis upon TMZ exposure. Upregulation of metalloproteinase inhibitor 3 (TIMP3) and inhibition of the PI3K/ACT signalling pathway and knockdown of MIF resulted in a significant increase in susceptivity of drug‐resistant glioma cells to TMZ. Exosomal MIF enhanced tumour growth and TMZ obstruction of glioma cells in vivo, while IOS‐1 (an MIF inhibitor) significantly increased glioma sensitivity to TMZ in vivo. Rehman et al. HSSP‐modified BMSCExo also delivered STAT3‐targeting siRNA to TMZ‐resistant gliomas and restored TMZ sensitivity. Chen et al. [109] found that miR‐27a‐3p presentation was higher in LN229r cells. miR‐27a‐3p‐silenced LN229r cells showed decreased IC50 values and proliferation rates and increased apoptosis rates. increased propagation of LN229r cells and decreased apoptosis could be achieved by EV treatment, while the opposite trend was observed for EVs silenced with miR‐27a‐3p. Thus, it is argued that GBM‐derived EVs are internalised by GBM cells and release miR‐27a‐3p into GBM cells, resulting in higher expression of miR‐ 27a‐3p, targeting BTG2 and improving TMZ obstruction. Jiang et al. [110] showed that LINC00473 explored the key role of TMZ in chemotherapy resistance and GBM. The results of qPCR analysis suggested that LINC00473 levels are raised in TMZ‐resistant cells after CREB activation, indicating that LINC00473 improves chemotherapy resistance in GBM cells by upregulating MGMT presentation. linc00473 binds to CEBPα to regulate MGMT expression and verified that exosomal LINC00473 transfers chemotherapy resistance from cell to cell and that high expression of LINC00473 in exosomes transfers chemotherapy resistance to GBM cells in the adjacent TMZ.

**TABLE 3:** Potential roles and mechanism of exosomes in the transmission of chemoresistance in glioma.

Molecular	Parent cell/source	Target cell	Target	Biological function	Reference
miR‐221	U87MG	SHG‐44	RELA/ miR‐221/ DNM3	Promote cell proliferation, migration, and TMZ resistance	[94]
miR‐151a	TMZ resistant GBM cells	TMZ sensitive GBM cells	miR‐151a/XRCC4	Inhibit TMZ resistance	[95]
miR‐1238	TMZ resistant GBM cells	TMZ sensitive GBM cells	miR‐1238/CAV1	Promote TMZ resistance	[96]
lncSBF2‐AS1	TMZ resistant GBM cells	TMZ sensitive GBM cells	ZEB1/SBF2‐AS1/miR‐151a‐3p/XRCC4	Promote TMZ resistance	[97]
circ‐HIPK3	TMZ resistant GBM cells	TMZ sensitive GBM cells	circ‐HIPK3/miR‐421/ ZIC5	Promote TMZ resistance and malignant progression of glioma	[115]
Cx43	TMZ resistant U251 cells	TMZ sensitive U251 cells	Cx43/ Bcl‐2/ Bax /Caspase‐3	Promote cell migration and enhance TMZ resistance	[98]
circ_0072083	TMZ resistant GBM cells	TMZ sensitive GBM cells	circ_007208/miR‐1252‐5/NANOG	Promote cell proliferation, migration, invasion, and xenograft tumour growth, as well as inhibit apoptosis and enhance TMZ resistance	[116]
miR‐25‐3p	A172R cells	TMZ sensitive GBM cells	miR‐25‐3p/FBXW7/c‐Myc/CCNDE	Promote cell proliferation and enhance TMZ resistance	[99]
MIF	TMZ resistant GBM cells	TMZ sensitive GBM cells	MIF/TIMP3/PI3K/AKT	Promote cell proliferation inhibit cell apoptosis and enhance TMZ resistance	[100]
miR‐27a‐3p	LN229r	TMZ sensitive GBM cells	miR‐27a‐3p/BTG2	Promote cell proliferation inhibit cell apoptosis and enhance TMZ resistance	[101]
circWDR62	TMZ resistant GBM cells	TMZ sensitive GBM cells	circWDR62/miR‐370‐3p/MGMT	Promote cell proliferation, migration, invasion, and enhance TMZ resistance	[65]
circ_0043949	TMZ resistant GBM cells	TMZ sensitive GBM cells	circ_0043949/miR‐876‐3p/ITGA1	Promote cell proliferation, migration, invasion, inhibit apoptosis and enhance TMZ resistance	[117]
LINC00473	TMZ resistant GBM cells	TMZ sensitive GBM cells	CREB/LINC00473/CEBPα/MGMT	Promote cell proliferation inhibit cell apoptosis and enhance TMZ resistance	[102]

**FIGURE 3:**
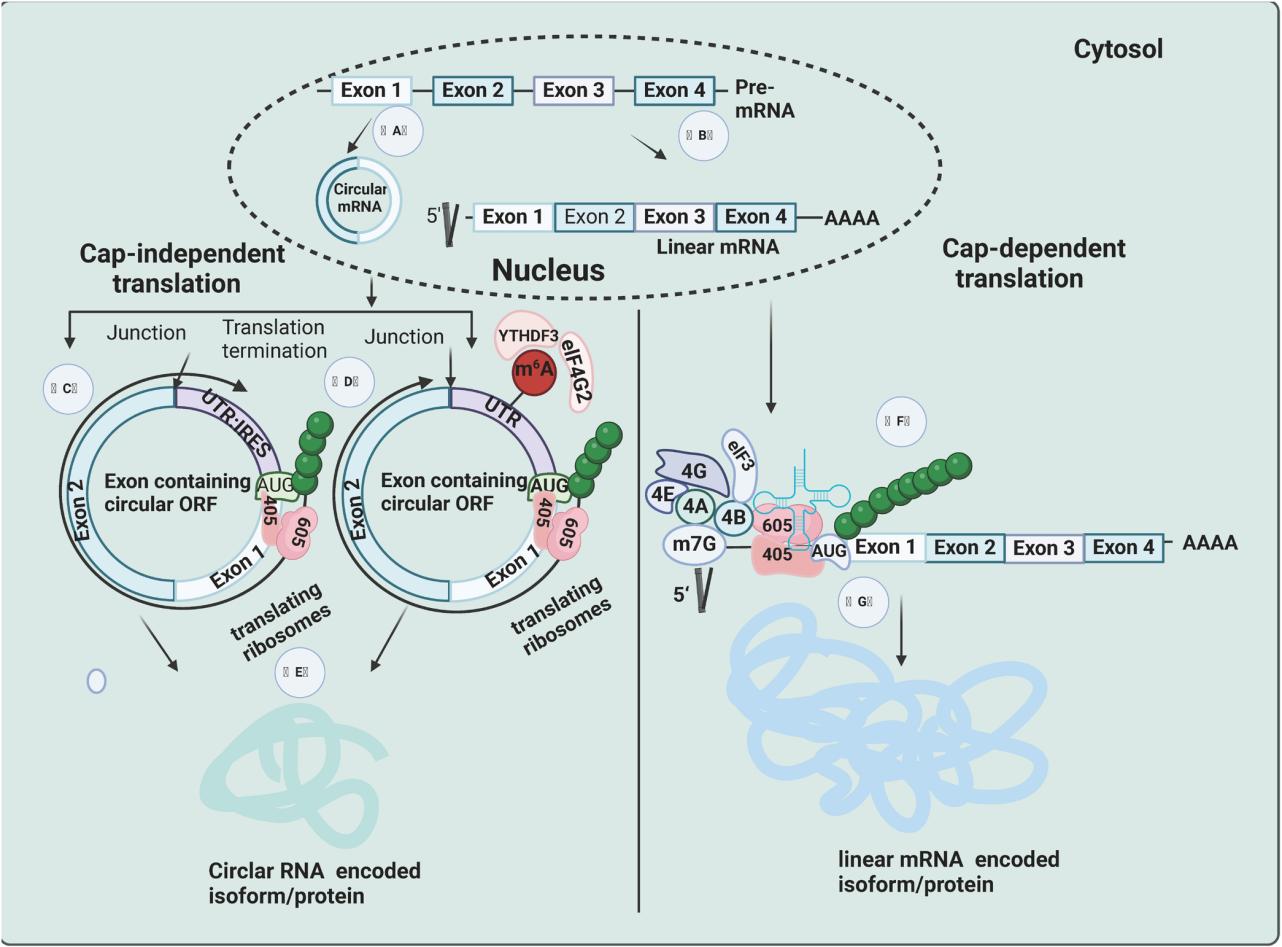
The canonical and non‐canonical translation processes of circular RNA and mRNA. (A) Non‐canonical reverse splicing of linear RNAs forms circular RNAs. (B) Typical alternate splicing leading to the production of linear mRNAs. (C) Internal ribosome entry site (IRES)‐mediated cap‐independent mechanism of circRNA translation. (D) Methylation of the sixth nitrogen in the adenosine in the UTR upstream of the AUG triplex of circRNAs in eukaryotic cells (m6A modification) initiated translation. (E) circRNAs encode novel isoforms or proteins with novel functions. (F) Typical 5′‐cap‐dependent mechanism of translational initiation of messenger mRNAs. (G) Processes by which linear mRNAs encode peptides/proteins.

Unlike traditional linear RNA, circRNA is a special type of non‐coding RNA without a 5′‐terminal cap and a 3′‐terminal poly (A) [111, 112]. The generation pathway of circRNA consists of three subclasses, and the most popular circRNA is exon‐derived circular RNA (ecRNA), which only contains exons and completely lacks introns [113, 114, 115]. Two mechanisms have been proposed for the biogenesis of ecRNA, including exon skipping and direct reverse splicing. During the process of exon skipping, downstream exons rotate and skip one or several exons to connect upstream exons, resulting in functional mRNA with exon skipping. At the same time, the jumping exons form a lasso precursor containing both exons and introns, which is then removed to form circRNA. The process of removing introns from the lasso is completed through standardised splicing. In contrast, direct reverse splicing first produces alternative splicing RNA and lasso intermediates, which are then more easily regulated by intron pairing mechanisms [116]. Recent studies have shown that direct reverse splicing may be the main mechanism for the formation of ecRNA. After biosynthesis, ecRNA must migrate to the cytoplasm in order to exert its regulatory effect. Another type of circRNA composed solely of introns, known as intron circular RNA (ciRNA), typically has the 3′ end of the exon shed into a loop [117]. It mainly exists in the nucleus and is mainly involved in regulating the transcription of its parent genes. CiRNA biogenesis requires a common motif, which consists of 7 nt GU rich elements near the 5′ splice site and 11 nt C rich elements near the branching site. This motif may be specifically involved in the formation of circRNAs, as it is not rich in conventional introns or other types of circRNAs. The biological process of ciRNA is regulated by its splicing mechanism mediated by eukaryotic spliceosomes. CiRNA is a circular intron that is cyclized at the 2 ′‐5′ linkage of the branching point and degraded from the 3′ end to the branching point. Therefore, they possess characteristics of resistance to branching and degradation, thus possessing high stability. Compared with other linear RNAs and non‐coding RNAs (ncRNAs), circRNA has several unique characteristics [118, 119, 120]. Most of these unique features are generated due to the presence of exons, while a small portion are generated due to introns and intron fragments. Generally speaking, the tissue‐specific and developmental stage specific expression patterns of circRNA are similar to the corresponding linear mRNA targets, with expression levels more than 10 times that of linear mRNA. At the same time, circRNA also exhibits evolutionarily conserved sequence characteristics across different species. And due to the lack of 5 ′‐3′ polarity in circRNA and its covalent closed loop structure without a polyadenylate tail, it helps to resist RNA exonuclease degradation. This is also why circRNAs are more stable than linear RNAs. CircRNA can exert stable biological effects because the average half‐life of circRNA in most species is longer than its linear counterpart [121, 122]. The canonical and non‐canonical translation processes of circular RNA and mRNA was showed in Figure 3.

Research has shown that exosomal circRNA also plays an important regulatory role in chemotherapy resistance in gliomas. Han et al. [123] demonstrated that circ‐HIPK3 could target miR‐421 and both were passively connected in glioma tissues. miR‐421 suppressed cell growth and TMZ resistance by overexpressing ZIC5. This experimental mechanism suggests that exosomal circ‐HIPK3 enhances cell advancement and TMZ resistance in TMZ‐resistant gliomas by regulating the miR‐421/ZIC5 axis. Ding et al. [124], proved that circ_0072083 knockdown of TMZ inhibited drug resistance in drug‐resistant cells by reducing IC50, propagation, transplantation, aggression, xenograft tumour raise and increasing apoptosis. Release of exosome circ_0072083 from drug‐resistant glioma cells was facilitated by the Warburg effect; rejection of subtle cells to TMZ was significantly enhanced by exosome circ_0072083 from drug‐resistant glioma cells. Exosome circ_0072083 is claimed to regulate miR‐1252‐5‐mediated degradation and demethylation, increasing NANOG and promoting glioma resistance to TMZ. Geng et al. [73] found that circWDR62 is transported between TMZ‐resistant and TMZ‐sensitive glioma cells through exosomes. circWDR62, an exosome of TMZ‐resistant cells, made receptor‐sensitive cells TMZ‐resistant and significantly increased the propagation, transplantation and aggression of these cells resulting in increased proliferation, transfer and aggression of these cells. The mechanism of this research argues that exosome‐mediated circWDR62 can promote TMZ resistance and virulent transformation of gliomas in vitro and in vivo by targeting the miR‐370‐3p/MGMT axis. Li et al. [125] showed that circ_0043949 was highly expressed in TMZ‐resistant GBM samples and cells, and suppression of circ_0043949 could reduce the IC50 of TMZ and decrease TMZ resistance. Circ_0043949 was enriched in TMZ‐resistant GBM cell‐derived exosomes and its expression was significantly upregulated in exosomes, and circ_0043949 in exosomes increased TMZ obstruction of TMZ‐resistant GBM cells in a xenograft model.

Exosomes play a central role in mediating chemotherapy resistance in gliomas through the transfer of various molecular components, including miRNAs, lncRNAs and circRNAs. These exosomal components regulate key molecular pathways, such as DNA repair mechanisms (e.g. XRCC4), apoptosis signalling and PI3K/AKT pathway activation, to promote drug resistance. The molecular pathways activated by exosomal components not only provide insight into the mechanisms of chemoresistance but also represent potential therapeutic targets for reversing resistance and improving treatment outcomes in glioma patients.

## 6 Exosome‐Based Therapies: Engineering and Delivery System Improvements in Glioma Treatment

To tackle the challenge of glioma chemoresistance, researchers have focused on engineering exosomes to improve their therapeutic potential. One promising strategy involves surface modification of exosomes. By attaching targeting ligands to the surface of exosomes, they can be directed specifically to glioma cells or areas of the tumour that are resistant to treatment. This targeted approach enhances the specificity of drug delivery and reduces the systemic toxicity often associated with conventional chemotherapy. In addition to surface modifications, improvements in exosome cargo loading have garnered significant attention. Exosomes can be loaded with a variety of therapeutic agents, such as siRNAs, chemotherapeutic drugs or gene‐editing tools. These engineered exosomes can deliver the drugs directly to tumour cells, bypassing barriers like the BBB and reducing the off‐target effects that often limit the efficacy of traditional therapies [126, 127]. For example, loading exosomes with siRNA targeting specific drug resistance genes in glioma cells could help reverse chemoresistance by silencing the expression of resistance‐associated proteins [128]. Furthermore, recent advances in optimising exosome production and purification methods are crucial for scaling up their clinical application. Standardising exosome isolation techniques and improving their stability will be essential for developing commercially viable exosome‐based therapies. As these technical hurdles are overcome, exosome‐based therapies hold great promise for enhancing the effectiveness of current treatment modalities in glioma and other cancers, providing a more precise, less invasive alternative to traditional chemotherapy.

By addressing the challenges related to exosome engineering, targeting and delivery, future research could lead to the development of exosome‐based therapies that not only overcome chemoresistance but also offer personalised treatment options for glioma patients. These innovations represent a significant step forward in cancer therapy, combining the precision of nanomedicine with the natural biological properties of exosomes.

## 7 Integration of Exosomal Profiling Into Clinical Practice for Glioma Treatment

Incorporating exosomal profiling into routine clinical practice, particularly for glioma patients undergoing chemotherapy, could offer several key advantages. One of the most exciting prospects is the use of exosomes as a form of liquid biopsy. Liquid biopsies, which involve sampling bodily fluids such as blood, CSF or urine, are non‐invasive methods that can provide real‐time insights into the molecular changes occurring in the tumour. Exosomal profiling from these fluids could be used to monitor the early development of chemoresistance, track therapeutic responses and even predict treatment outcomes. For instance, by analysing exosomal miRNA or protein signatures, clinicians may be able to identify whether glioma cells are acquiring resistance to common chemotherapeutic agents, such as TMZ [129]. This could inform adjustments in treatment plans, ensuring that patients receive the most effective therapies based on the specific molecular landscape of their tumours. Moreover, exosomal biomarkers could enable the detection of minimal residual disease, helping to monitor remission and detect potential relapse before clinical symptoms appear. Integrating exosomal profiling into clinical decision‐making could also support the move toward more personalised treatment strategies [130]. Since exosomes carry cargo reflective of the tumour's genetic and epigenetic landscape, their analysis could provide valuable insights into the heterogeneity of gliomas and the individual molecular mechanisms driving drug resistance in each patient. This personalised approach would allow for more precise targeting of therapeutic interventions, improving both patient outcomes and quality of life.

However, translating exosomal profiling from laboratory research into clinical practice is not without its challenges. Issues related to standardisation of exosome isolation and characterisation methods, as well as the need for large‐scale clinical validation studies, must be addressed. Additionally, regulatory considerations around the use of exosome‐based diagnostics and therapies need to be carefully navigated to ensure safety and efficacy.

## 8 Discussion and Prospects

Cancer is not only caused by a great number of virulent cells, but is also a tangle some systemic manifestation in which various cell kinds are involved in tumour homeostasis, including fibroblasts, adipocytes, immune cells and cells of the tumour vasculature [28, 131]. Antitumor resistance due to various physiological acts has long been an interference to tumour therapy [132]. Despite significant advances in antitumor drugs, the development of rejection often contributes to failure of tumour therapy. Excepting for living chemotherapy and new immunotherapies, there is an exigent need to exploit new approaches to remove tumour advancement and recurrence due to drug resistance. Chemoresistance is also a common trouble in the cure of gliomas, where tumours do not shrink or continue to grow despite the patient receiving several cycles of chemotherapy. There are many reasons for chemotherapy resistance, including natural resistance of tumour cells to chemotherapy drugs, insufficient drug concentrations in tumour tissue and increased drug metabolism by tumour cells. Current research suggests that chemotherapy resistance in gliomas is due to problems with drug absorption, transport, metabolism and excretion by tumour cells [133, 134]. In addition, factors such as genetic mutations in tumour cells, abnormalities in apoptotic mechanisms, and alterations in the tumour microenvironment may also affect sensitivity to chemotherapeutic agents [135, 136]. To overcome chemotherapeutic drug resistance in gliomas, new chemotherapeutic agents can be developed or more effective drug combinations can be found to improve tumour cell sensitivity to the drugs. In addition, targeted therapeutic approaches such as gene therapy and immunotherapy can also be used to address various components of the above mechanisms.

Exosomes have been found to play a prominent part in mediating resistance to tumour chemotherapy by delivering contents such as nucleic acids and proteins. The mechanisms by which exosomes mediate tumour resistance to chemotherapy are primarily revealed by acting on recipient cells, for example, by inducing the formation of a premetastatic ecological niche [137]. They are also mediated by decreasing intracellular drug concentrations, for example, by increasing drug efflux. Exosomes can also shift drug‐resistant phenotypes from drug‐resistant cells to vulnerable cells [138]. In addition, exosomes can modulate tumour drug resistance by restructuring the tumour microenvironment, including increasing tumour cell immune escape and promoting tumour angiogenesis. Exosomes have the distinct underlying to seize the actional sophistication of cancer and can be used to detect various biological parts linked to tumour drug resistance real‐time, but current comprehending of exosome physiology, release, transport, internalisation and transport mechanisms is finite, and more comprehensive studies of the interaction and alteration mechanisms between exosomes and receptor cells are needed. Despite these challenges, exosomes may be used as candidate biomarkers for forecasting and supervising therapeutic efficacy in tumour patients and as possible targets or vectors for reversing drug resistance, and will play a crucial character in the exploration, forecast and remedy of future tumour It will play an prominent character in the exploration, forecast and remedy of future tumours.

The burgeoning domain of combinatorial therapy holds significant potential, especially through the utilisation of exosomes‐microscopic vesicles‐as vectors for immune‐modulating agents. These exosomes can be engineered to deliver immune checkpoint inhibitors or other immunotherapeutics, potentially enhancing the immune system's ability to detect and eradicate glioma cells. For instance, exosomes equipped with PD‐1/PD‐L1 inhibitors might disrupt the mechanisms tumours use to circumvent immune surveillance. This precision‐targeted approach ensures the direct administration of therapeutics to the tumour site, thereby augmenting immunotherapy efficacy and mitigating adverse effects [139]. Additionally, exosome‐based modalities can be synergistically paired with other targeted treatments for increased therapeutic potency. Gliomas often exhibit distinctive genetic aberrations that can be precisely targeted. By bioengineering exosomes to carry siRNAs or CRISPR‐Cas9 systems, we can potentially suppress or modify genes linked to chemoresistance, thus rendering tumour cells more susceptible to chemotherapy and possibly reversing resistance phenotypes.

A paramount challenge in glioma treatment is the BBB, which impedes the delivery of many pharmacological agents to the brain [140]. Exosomes, due to their nanoscale dimensions and ability to traverse biological barriers, offer a promising solution. Designed to convey chemotherapeutic agents or genetic payloads, they can efficiently deliver these compounds to glioma cells within the brain, thereby overcoming BBB limitations and enhancing drug bioavailability [141]. To successfully integrate exosome‐based combinatorial therapies into clinical practice, several hurdles must be surmounted, such as standardising exosome isolation, ensuring reproducibility and addressing regulatory concerns. Advances in high‐throughput sequencing and proteomics will be critical for analysing exosomal content and understanding their roles in drug resistance and immune modulation. In summation, the amalgamation of exosome‐based strategies with combinatorial therapies offers a comprehensive approach to overcoming glioma drug resistance and improving patient outcomes [142]. By leveraging and customising the intrinsic properties of exosomes, we can develop more effective, targeted and personalised therapeutic strategies for glioma patients, potentially ushering in a paradigm shift in the current glioma treatment paradigm as research continues to advance [143].

While preclinical studies have demonstrated that exosomes can carry chemoresistance‐related biomarkers and therapeutic molecules, there is a lack of ongoing clinical trials that aim to use exosomes in real‐world settings for glioma patients. This research gap is a notable shortcoming, as it prevents the translation of laboratory findings into practical, clinical applications. Furthermore, the clinical validation of exosomal biomarkers, such as miRNAs or proteins associated with chemoresistance, is still in early stages. Until larger, well‐structured clinical trials are conducted, the potential of exosomes in managing glioma chemoresistance will remain largely theoretical. The absence of focused clinical trials also highlights the broader challenge of bridging the gap between benchside discoveries and bedside applications in the field of cancer therapeutics. Moving forward, clinical trials that explore exosome‐based therapies and diagnostic tools for gliomas need to be prioritised. These trials should focus on not only the efficacy of exosome‐targeted drug delivery systems but also on their role in identifying patients likely to respond to specific chemotherapies based on exosomal molecular signatures.

This paper outlines the biological role and potential molecular mechanisms of exosomes in adjusting tumour drug resistance in gliomas. To date, however, studies on exosomes and glioma drug resistance have been mainly limited to the in vitro level, and no relevant studies in clinical cases have been reported. Translating experimental results at the cellular level into clinical trials remains the most challenging task. On the one hand, the prospect of using exosomes therapeutically, which mainly involves the exchange of extracellular information and the control of targeted delivery of drugs, requires more detailed studies to clarify the specific mechanisms of exosome secretion and delivery. Meanwhile, the delivery efficiency of exosomes needs to be further improved.

## 9 Conclusion

This review focuses on the molecular mechanisms of exosome‐mediated chemotherapy resistance in glioma, but also recognises that research on exosomes and chemotherapy resistance is still in its early stages. Detailed studies of the molecular mechanisms of exosome‐mediated chemoresistance in glioma will be of great significance for the development of new chemotherapeutic agents, enhancement of glioma cell sensitivity to chemotherapeutic agents, and improved survival of glioma patients.

## Author Contributions

**Xu Guo:** writing – original draft (equal), writing – review and editing (equal). **Haozhe Piao:** writing – review and editing (equal). **Rui Sui:** writing – original draft (equal).

## Ethics Statement

The authors have nothing to report.

## Consent

The authors have nothing to report.

## Conflicts of Interest

The authors declare no conflicts of interest.

## Data Availability

The data that support the findings of this study are available from the corresponding author upon reasonable request.
